# Proceedings of Medical Societies

**Published:** 1848

**Authors:** 


### ARTICLE VIII.

### *Proceedings of Medical Societies communicated for Publication.*

*The Upper Wabash Medical Association.*—Pursuant to previous notice, about twenty physicians, from the counties of Cass, Miami, Wabash, &c., assembled at Wabash, Wabash county, on Wednesday, May 10th, in Medical Convention. Professor G. N. Fitch, of Logansport, was appointed President, and Dr. James Ford, of Wabash, Secretary.

On motion,

*Resolved*, That this Convention form an association, to be known by the name of "*The Upper Wabash Medical Association.*"

*Resolved*, That the President of this Convention, appoint a committee of three to prepare a Constitution, By-Laws, and Code of Medical Ethics, to be submitted to the next meeting of the Convention.

C. V. N. Lent, U. Farquahar, and James Ford, were appointed such committee.

On motion,

The following instructions were sent to said committee:

"To report as necessary for a membership of the Association :—A good English education, a good moral character, a diploma from some regular college, or in the absence of a diploma, a license from any regular Medical College, or from any duly organized society of regular physicians—or, if the candidate has neither diploma or license, or, a certificate that he has read medicine three years under any regular physician of good standing, or been a respectable practitioner of medicine, for three years, and is possessed of the two first requirements, shall entitle him to an examination by the Board of Censors of the Association, for admission to a membership."

*Resolved*, That a committee of three be appointed, to examine into the credentials of those who shall offer themselves for a membership of the Association.

R. Faber, A. Chapman, and C. V. N. Lent, were appointed such committee.

*Resolved*, That a committee of two or more, from each county within the limits of the Association, be appointed to present at the next meeting, or as early thereafter as possible, reports of the medical topography of their respective counties, and that they be instructed to embrace in their report, especially, the following matters, viz: Streams, their size, current, quality of water, duration, beds, banks, whether dry or low and marshy; soil, timber, its quality and quantity; surface, level or undulating; prairie, quantity and quality; mineral deposits; rock, what kinds and quality; ponds and lakes, their size, quantity of water, nature of shores, &c.; medical botany; any cause producing or modifying disease; prevalent diseases.

The following committee was appointed under this resolution, viz.:

*Grant county.*—Wm. Lomax and J. S. Shively.

*Wabash county.*—Jas. Ford, S. A. Barry, and —— Mussy.

*Miami county.*—E. H. Salter, G. C. Paramore, J. S. Cole, and J. H. Constant.

*Fulton county.*—Charles Brackett and J. J. Shryock.

*Howard county.*—C. Richmond and J. H. Kerns.

*Cass county.*—G. N. Fitch, R. Faber, and A. B. Buchanan.

*Resolved*, That R. Faber be requested to prepare himself by the next meeting of the Convention, for keeping from and after that time, a Meteorological Table for the use of the Association.

*Resolved*, That the President and Secretary, of this Convention, be requested to deliver essays on some medical subject, at the next meeting of the Convention.

*Resolved*, That this Convention meet on Wednesday, 21st June, at 10 o'clock, A. M., at the Western House, in Peru, for the purpose of perfecting the organization of the Association, and otherwise carrying out the designs of the resolutions of this Convention.

*Resolved*. That the Physicians of Huntington, Wabash, Miami, Grant, Cass, Fulton, Howard, Carroll, and Pulaski counties, be invited to meet and unite with us at that time and place.

*Resolved*, That the Editors of all the newspapers in the above counties, be requested to publish the proceedings of this Convention, and that a copy of the same, be sent to the North-Western Medical and Surgical Journal, (late Indiana and Illinois Medical and Surgical Journal.)

The Convention then adjourned, to meet again as resolved.

G. N. FITCH, *President*.

JAMES FORD, *Secretary*.

*Proceedings of Rock River Medical Society, May* 16*th*, 1848, *at Dixon, Ill.*—The meeting was called to order by the President, J. B. NASH, M.D. Dr. OLIVER EVERETT, was chosen Vice President, *pro. tem.* JOS. S. LANE, M.D., Secretary, *pro. tem.*

The following gentlemen were admitted members of the Society: Drs. G. W. Chittenden, J. J. Baten, Wm. O. Chamberlain, S. Allen Paddock, Ephraim Ingals, and A. S. Hudson.

The President then delivered an address to the Society, on the causes of our western diseases.

After some discussion, on different subjects, the Society then proceeded to the election of officers, which resulted in electing the following gentlemen:

*President.*—L. CLARK, M.D., Rockford, Ill.

*1st Vice President.*—Jos. S. Lane, M.D., Janesville, Wis.

*2d Vice President.*—Wm. O. Chamberlain, M.D., Princeton, Illinois.

*Treasurer and Secretary.*—A. M. Catlin, M.D., Rockford, Ill.

*Censors.*—W. W. Welch, M.D., Inlet Grove, Ill.; Oliver Everett, M.D., Dixon, Ill.; Jos. S. Lane, M.D., Janesville, Wisconsin.

The President appointed Dr. G. W. Chittenden and Dr. A. Clark, to deliver addresses before the Society, at its semi-annual meeting. Also, Dr. A. W. Benton and Dr. C. Martin, to deliver addresses before the Society, at its next annual meeting.

The following resolutions were then adopted:

*Resolved*, That the thanks of this Society are due, and are hereby tendered to our President, Dr. J. B. Nash, for his interesting and instructive address, delivered before it.

*Resolved*, That the annual meeting be held at Rockford on the 3d Tuesday of May.

*Resolved*, That the semi-annual meeting be held at Janesville, Wisconsin, on the 1st Tuesday in November.

The Society then adjourned.

JOSEPH S. LANE, *Secretary.*

*Fort Wayne Medical Society.*—This Association was organized in the city of Fort Wayne, on Tuesday, May 2d, 1848, and is to be composed of the regular practitioners of medicine, in Allen and the adjoining counties.

The following officers were elected for the ensuing year, viz.:

*President.*—Dr. Thomas Hamilton, Lagro, Ia.

*Vice President.*—Dr. C. E. Sturgis, Fort Wayne, Ia.

*Treasurer.*—Dr. J. M. Kitchen, Fort Wayne, Ia.

*Secretary.*—Dr. S. S. Thompson, Fort Wayne, Ia.

A Board of Censors was chosen, for the examination of applicants for membership. The Society meets again, on Tuesday, 7th Nov. next, at the Court House, in Fort Wayne.

*Western Medical Society of Wayne county, Indiana.*—At a meeting of the Western Medical Society, of Wayne county, Indiana, held in Hagerstown, on the first Monday in March last, it was "resolved that the President and Secretary, be requested to inform the Editors of the North-Western Medical and Surgical Journal, of the organization of this Society; and that each member be requested to subscribe for one copy of said Journal."

This Society was organized on the sixth of October, 1846. Numbers about twenty members. It holds its stated meetings on the first Monday of March, June, September, and December, at such places as the Society adjourns to. It elects its officers annually, at its meetings in June.

N. JOHNSON, *President*.

AUGUSTUS WEAVER, *Secretary*.

*Indianapolis Medical Society*.—The Society held its regular session, on Saturday evening, June 3d.

Dr. Livingston Dunlap read a highly interesting paper, on the nature and treatment of hydrocele.

A discussion followed, on the merits of the operations performed, and the pathology of the disease. This was very generally participated in, by the members, and was highly interesting.

After transacting some local business, the Society adjourned to the next regular meeting.

J. S. BOBBS, *Secretary*.

